# Synchronization and Coordination of Art Performances in Highly Competitive Contexts: Battle Scenes of Expert Breakdancers

**DOI:** 10.3389/fpsyg.2021.635534

**Published:** 2021-04-15

**Authors:** Daichi Shimizu, Takeshi Okada

**Affiliations:** Graduate School of Education, The University of Tokyo, Tokyo, Japan

**Keywords:** performing arts, interpersonal coordination, synchronization, breakdance, relative phase, competitive context

## Abstract

In the performing arts, such as music and dance performances, people actively interact with each other and show their exciting performances. Some studies have proposed that this interaction is a social origin of the performing arts. Some have further investigated this phenomenon based on the synchronization and coordination theory. Though the majority of these studies have focused on the collaborative context, several genres of the performing arts, such as jazz sessions and breakdance battles, have a competitive context. Several studies have suggested that, in this competitive context, performers actively interact with each other and construct some correspondence. Moreover, a few recent studies focusing on competitive conversations, such as debates, have shown that, compared to people's interactions in collaborative conversations, people in competitive contexts frequently coordinate their behaviors in complicated ways. However, the interaction and coordination among performers in these competitive contexts have not been sufficiently investigated. Therefore, we investigated the coordination of expert breakdancers in battle scenes and measured their rhythmic movements using a motion capture system. We calculated the relative phase of the rhythmic movements between two dancers to investigate their coordination. The results showed that the dancers' rhythmic movements tended to synchronize in an anti-phase fashion, which means that there were similarities as well as differences between the two dancers' rhythmic movements. Furthermore, this pattern of coordination changed dynamically as time elapsed, from an in-phase synchronization or leader-follower relationships to an anti-phase synchronization and then leader-follower relationships.

## Introduction

In the domain of the performing arts, which includes dance, theater, and music performances, performers actively interact with each other while showing interesting performances (Bailey, 1980; Merker et al., 2015). Some performers and researchers have described this interaction as an essential factor in the performing arts (Bailey, 1980) because such a complicated interaction among performers attracts the interest of the audience.

Additionally, some theories have proposed that this interaction plays a vital role in human society (Fitch, 2006; Merker et al., 2009; Kirschner and Tomasello, 2010; Ravignani et al., 2014) because it has the function of building and facilitating social bonds among people, which contributes to the maintenance and development of a community. As people benefit from living in a large group (Dunbar, 1998), music performances and dances have spread across almost all cultures and communities. Recent studies have quantitatively suggested that people's relationships strengthen when they collectively participate in music or dance performances (Wiltermuth and Heath, 2009; Kirschner and Tomasello, 2010; Weinstein et al., 2016).

In recent years, several researchers have attempted to investigate this complicated interaction by applying the synchronization and coordination theory (e.g., Ravignani et al., 2014; Washburn et al., 2014; Walton et al., 2015, 2018). Some studies suggest that the synchronization and coordination theory originated in the observations of the physicist Huygens on clock pendulums (Huygens, 1669; Bennett et al., 2002; Ramirez et al., 2016). It has been applied theoretically and empirically in physics, the dynamical systems approach (Schmidt et al., 1990; Strogatz, 2003), and biology (Jones, 1964; Buck and Buck, 1966). In recent years, psychology researchers have applied it to investigate behavior correspondence among multiple individuals (Bernieri and Rosenthal, 1991; Keller et al., 2014). Since different research domains use different definitions, it is not easy to set a clear definition. Nonetheless, based on the definition from and the features of applied phenomena in psychology, the current study defines “synchronization” as the periodic repetition of similar actions with matched timing, like in-phase synchronization and anti-phase synchronization (e.g., Bernieri and Rosenthal, 1991; Fujiwara and Daibo, 2016). We additionally define “coordination” as relating to the broader range of behavior correspondences—like leader-follower relationships and polyrhythms—in addition to synchronization (Konvalinka et al., 2010). In these correspondences, similar actions that show deviations in timing (leader-follower relationships) or period (polyrhythms) are observed among individual behaviors.

A representative example of the synchronization and coordination is the group behaviors of insects and anurans (Buck and Buck, 1966; Fitch, 2006; Merker et al., 2009; Ravignani et al., 2014). They offer interesting suggestions concerning the performers' interactions. Studies focusing on these creatures have suggested that the timings of the flashing of fireflies and the choruses of anurans, crickets, and katydids match up strongly within the group when they gather (in-phase synchronization). Some studies have explained that this in-phase synchronization not only attracts distant heterosexuals and increases their chances of mating but also disrupts their predators and decreases their risk of being eaten or killed (Ryan et al., 1981; Ravignani et al., 2014).

However, other studies have observed another interesting behavior in some groups of insects (Jones, 1964; Walker, 1969). They showed that some species of crickets and katydids often chirp alternately, rather than with the same timing. These species actively change their leader-follower relationships of chirps as if they are competing. Several studies have proposed that the females of these species prefer the individuals that chirp ahead (Greenfield and Roizen, 1993; Snedden and Greenfield, 1998; Grafe, 1999; Greenfield et al., 2017), suggesting that the leader has a higher chance of mating. These studies suggested that groups of insects show different group behaviors depending on their situations and contexts (collaboration or competition within the groups). If many individuals of the species can gain significant benefits from mating and decrease their risk of becoming a prey from collaboration, they will show an intense synchronization across their chirps. If many individuals of the species can gain higher chances of mating from competition, they will chirp alternately. Greenfield et al. (2017) differentiated these phenomena as perfect synchrony and alteration, and they sought to investigate each mechanism.

As what these studies of insects have suggested, the situation and context of collaboration/competition have an essential influence on the interaction process among individuals. However, the studies investigating the performers' interactions have mostly focused on the collaborative context in which several performers try to achieve a common goal, as in the achievement of a single structured and attractive performance. For example, Walton et al. (2015, 2018) investigated the behaviors of two pianists in their improvisations and showed that their hand movements tended to move in an in-phase synchronization. Furthermore, Kimmel and Preuschl (2016) investigated the behaviors of tango dance pairs and showed that a pair's head movements tended to move in an in-phase synchronization. These studies provided important suggestions about the coordinated behaviors among performers in a collaborative context. However, the interactions among performers in a competitive context, such as jazz sessions and breakdance battles, have not been sufficiently investigated. In these genres and situations, performers compete against each other to try and show impressive individual performances that are better than those of their co-performers. Several studies have suggested that this competitive context generates complicated interactions among performers, which attract audiences (Bailey, 1980; Shimizu and Okada, 2013). We observed that expert dancers mimicked and further developed the expressions of other dancers in their performance scenes (Shimizu and Okada, 2013). However, this complicated interaction among performers has not yet been sufficiently investigated from a scientific perspective. Therefore, this study investigates the performers' interactions in a situation that facilitates competition among them by applying the synchronization and coordination theory.

Studies that have investigated the synchronization and coordination of human species in a competitive context are limited (e.g., Paxton and Dale, 2013, 2017; Abney et al., 2014). Several studies have investigated the coordination in conversations. For example, Paxton and Dale (2013, 2017) compared people's behaviors of the body in collaborative conversations (conversations about their favorite things, such as TV shows and music) with those in competitive conversations (debates on social issues). They showed that the time-lagged coordination of the movements of the head and whole bodies, which is similar to alteration and anti-phase synchronization, was frequently observed in the competitive context. Furthermore, they indicated that the in-phase synchronization of these head and body movements was less observed in the competitive context than in the collaborative context. These results suggested that in the competitive context, people harmonize some aspects of their behaviors of the body, such as the movement frequency, while simultaneously differentiating some other aspects, such as the movement timing within the group. These results are consistent with studies focusing on the interaction of insects in the competitive context, which showed the time-lagged coordination of chirps (Jones, 1964; Walker, 1969).

Moreover, Keller et al. (2017) investigated the group behaviors of a traditional boys' chorus in Germany and offered a similar suggestion. This study first created a situation wherein girls in puberty demonstrated their appreciation of the boys' singing in order to strengthen the competitive context. The researchers compared the boys' singing in this situation with their singing in other situations. The results showed that, in this competitive context, the boys with the lowest singing voice (Bass part) emphasized their individual voices while keeping in harmony with the other parts (Soprano, Alto, and Tenor). Though the singers did not intentionally change their behaviors, they evaluated the performances that received appreciation from the girls as the ones with the highest quality. These results suggested that the performers' interactions in a competitive context showed some interesting features. The performers tried to emphasize their individual performances while matching some aspects of their performances with those of others.

Based on these studies, this study quantitatively investigates the coordination among performers in the competitive context by applying the synchronization and coordination theory. Primarily, we focus on a breakdance battle scene in which two dancers show their performances in turn. After which, the judges selected the winner (OHJI, 2001; Watkins, 2005). In a breakdance battle scene, dancers perform various domain/original dance steps while simultaneously conducting a rhythmic movement called “Up.” In this “Up” movement, dancers take their rhythms by flexing and extending their knees repeatedly. While various ways of rhythm-taking are possible depending on the dancers' individuality and music features, the most basic way is to match the knee extension's timing to the music beat.

Furthermore, in a battle scene, dancers actively interact with each other while performing these rhythmic movements and dance steps. They frequently cite and develop another dancer's dance steps and rhythmic movements. Additionally, they often make several provocative gestures during their dance steps, and when they watch another dancer's performance in that dancer's performance turn, they actively interact and compete by using gestures, facial expressions, and rhythmic movements. We investigate whether this interaction in the competitive context shows a specific type of synchronization and coordination. Based on the findings of previous studies, we hypothesize that battle scene dancers show an *anti-phase synchronization*, or behaviors in which some aspects match up with one another while others differ, like coordination in a competitive conversation. Furthermore, we hypothesize that dancers exhibit leader-follower relationships in some interaction phases, in ways that are similar to the leader-follower relationships (and the dynamic changes therein) seen among insects (Walker, 1969).

In this study, we examine the relative phase angles of the dancers' rhythmic movements (the coordination of the rhythmic movements between two dancers). Several studies have suggested that rhythmic movement is the foundation of dance expression (Miura et al., 2011). Additionally, in the breakdance domain, the dancer's rhythmic movement is one of the essential factors of their expression (OHJI, 2001; Watkins, 2005). In a famous evaluation system called the O. U. R. System, the dancers' rhythm in their performances is one of the five most heavily weighted values. However, in the battle scene, there is no obvious rule about the rhythmic movements of two dancers. They can adopt a rhythm freely in the battle scene (Watkins, 2005). These discussions have suggested that rhythmic movement is an appropriate measure to investigate the coordination of dancers in a breakdance battle scene.

## Materials and Methods

### Participants

Seven expert breakdancers participated in our study (Expert A: 26 years old, 8 years' experience; B: 28 years old, 12 years' experience; C: 23 years old, 7 years' experience; D: 29 years old, 13 years' experience; E: 29 years old, 13 years' experience; F: 26 years old, 10 years' experience; G: 30 years old, 13 years' experience). All experts had the experience of winning first or second prizes in breakdancing competitions held in Japan. The experts were divided into two groups (group 1: A, B, C, D; group 2: B, E, F, G), and each group participated in the experiment separately.

### Procedures

**Figure 1:**
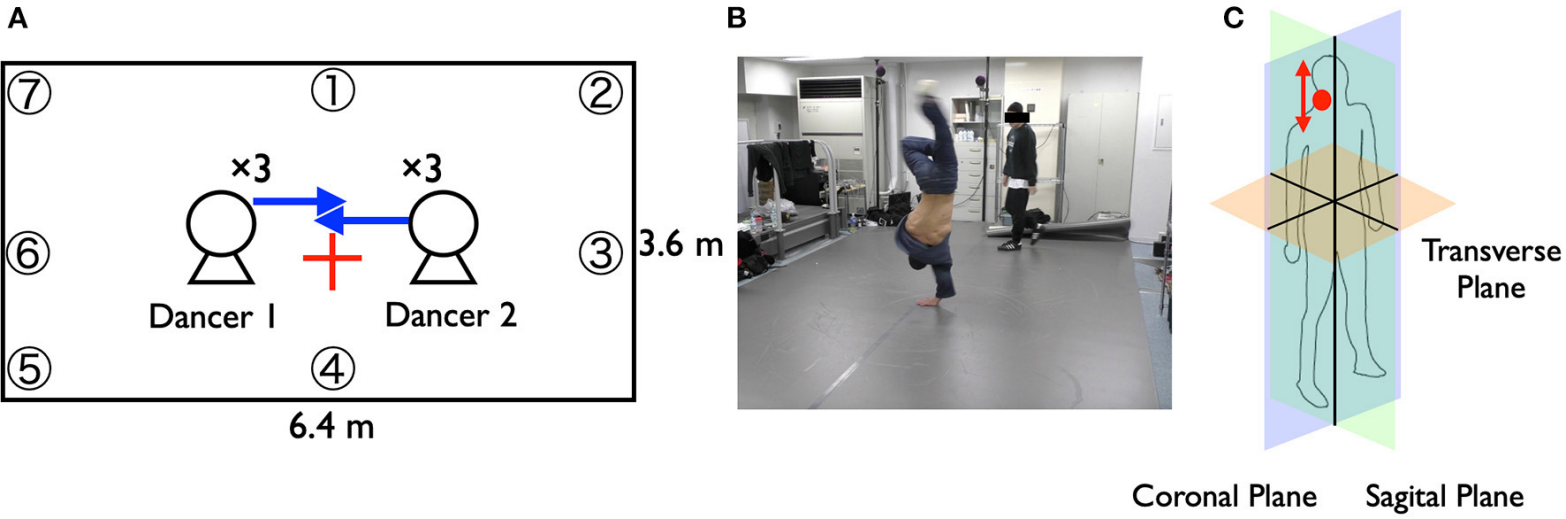
**(A)** Outline of the battle scene. Two dancers faced each other and showed their performances in turn. **(B)** Picture of the battle scene. One dancer showed his performance, and another dancer watched and responded to his performance. **(C)** Body plane. The red circle indicates the marker position, and the red arrow indicates the movement direction we analyzed.

Four expert dancers in each group paired up and conducted the battles in a round robin tournament (six pairs in each group), meaning that 12 battles were conducted. Each battle allowed each dancer to perform three times in turn (see Figures 1A,B). We excluded the data from three battles from the analysis because of a malfunction of the system and because of some missing markers, which meant that we could not adequately measure the dancers' positions. In our future work, we aim to invent methods and devices to measure the dancers' dynamic and acrobatic movements sufficiently in the experiments and in more natural situations for fieldwork.

We set the experimental situations as close to real battle scenes as possible (OHJI, 2001; Watkins, 2005). Therefore, we did not decide the order of their performances in advance. The dancers decided the order through their interactions in the battle. We also did not set any time limits to their performances or instruct the dancers about the kind of music to use in their performances. We used the same music in all the battles (DJ Fleg “Chelles”).

**Figure 2:**
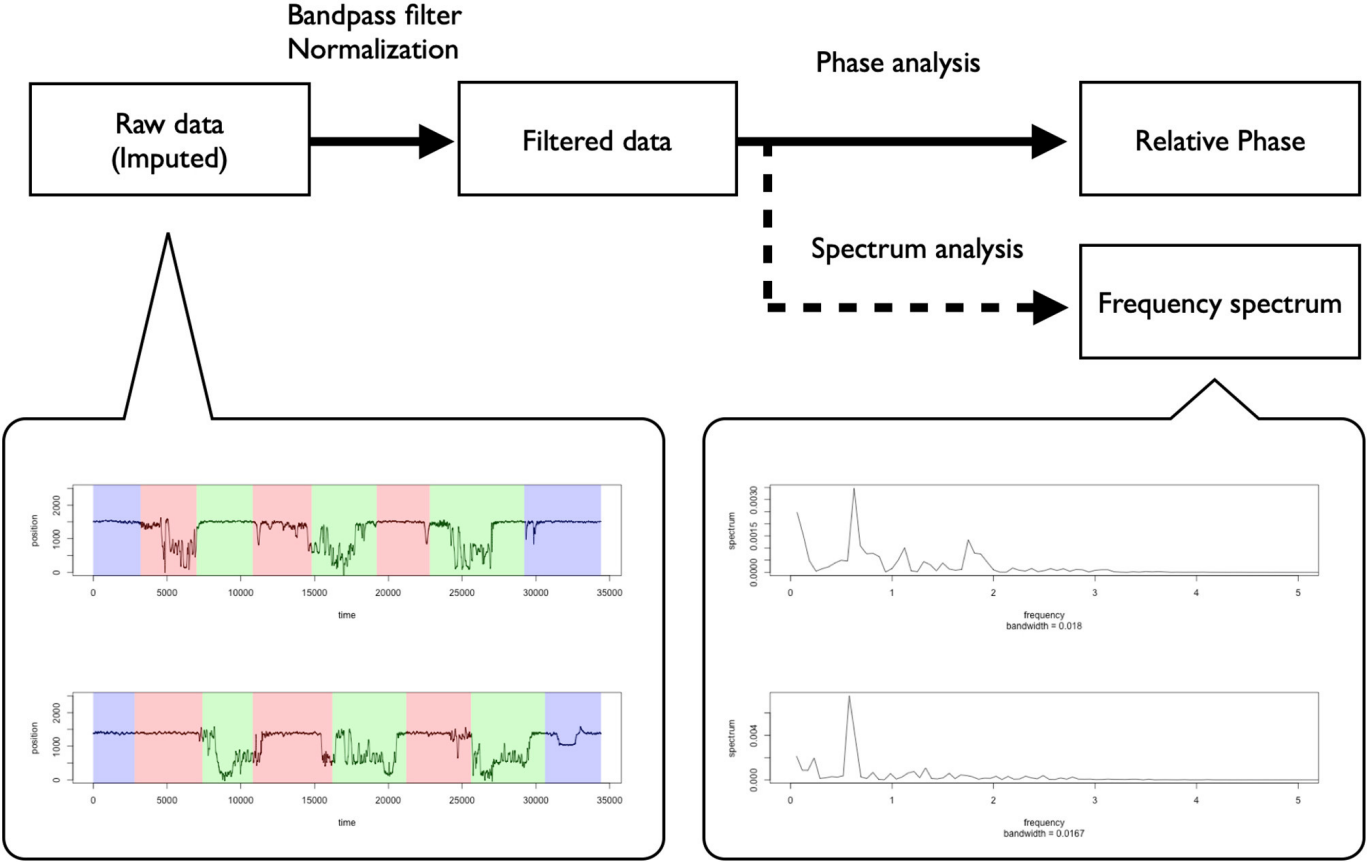
Normalization procedures and the representative examples of raw data (Whole turns) and spectrum analysis (Turn 1) of individual-level rhythmic movement data.

We measured the movement data of each dancer using an infrared motion capture system (OQUS 300, QUALISYS, Göteborg, Sweden). For the movement measurements, we put seven markers on each dancer in locations where the markers did not interrupt their performances based on a pilot study (five around their necks and two on their trunks). We used, in the analysis, the markers' z-axis movement data around their necks (Figure 1C). The necks' vertical movements seem to represent the essential features of the dancers' rhythmic movements in breakdance because, as discussed in the introduction, dancers adapt their rhythms by moving their whole bodies upward/downward. We used R (3.5.2) to analyze the data, imputing the movement data's missing values via spline interpolation and smoothing them using a bandpass filter (1–5 Hz). We set this band while considering the predicted frequency of the basic rhythmic movements described in the introduction. Additionally, applying the high-pass filter is one of the preferred methods by which to exclude data trends and calculate the appropriate phase (de Poel et al., 2020). After smoothing and adjusting the data for phase analysis, we applied a typical normalizing procedure to subtract the whole mean and divide by the standard deviation. Figure 2 summarizes these procedures.

### Ethics Statement

The experimental procedures were conducted in accordance with the Declaration of Helsinki and approved by the Ethics Committee of the University of Tokyo. All dancers provided a written informed consent and were paid for their participation.

### Analysis

**Figure 3:**
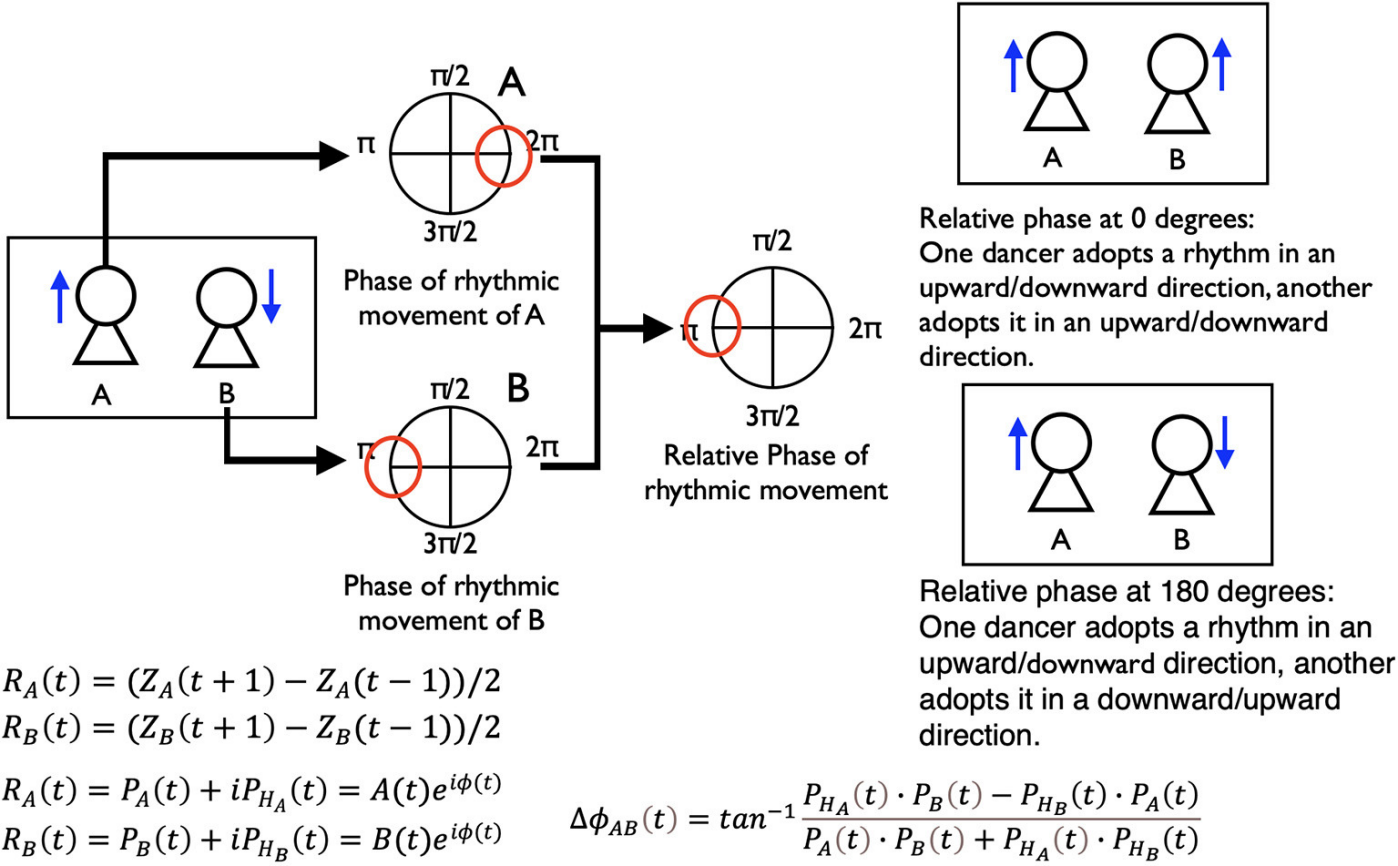
Procedures of calculating the relative phase of the two dancers' rhythmic movements. We made this explanation by referring to Kijima et al. (2012) and Okumura et al. (2012).

We calculated the relative phase of the rhythmic movements between two dancers using the z-axis data of the above markers. We conducted these analyses by developing a method based on several studies that investigated the coordination of movements in the space between two players in Kendo matches and a game of tag (Kijima et al., 2012; Okumura et al., 2012). There are many kinds of analyses by which to investigate the synchronization and coordination of people's behaviors. However, the current study is an exploratory one, which is undertaken to quantitatively capture the complicated human interactions within a performance. We thought that it was best to start with a simple method to calculate the coordination of rhythmic movements, and thus selected the following method. We detected the relative phase of the rhythmic movements between two dancers using the following three steps: (1) calculate the vector of the rhythmic movements; (2) use the Hilbert transformation to calculate a phase of each dancer's rhythmic movements; and (3) use the above phase data of each dancer to calculate the relative phase between two dancers (Figure 3). (1) First, we calculated the vector of the dancer's rhythmic movements (movements of the z-axis direction) in time t and normalized these vector data. R_A_ is the vector of the rhythmic movements of one dancer and R_B_ is that of another dancer.

$$\begin{array}{l} R_{A}\left(t\right)=\left(Z_{A}\left(t+1\right)-Z_{A}\left(t-1\right)\right)/2\\ R_{B}\left(t\right)=\left(Z_{B}\left(t+1\right)-Z_{B}\left(t-1\right)\right)/2 \end{array}$$

(2) Second, we used the Hilbert transformation to calculate a phase of each dancer's rhythmic movement vector.

$$\begin{array}{l} R_{A}\left(t\right)=P_{A}\left(t\right)+iP_{H_{A}}\left(t\right)=A\left(t\right)e^{i\phi \left(t\right)}\\ R_{B}\left(t\right)=P_{B}\left(t\right)+iP_{H_{B}}\left(t\right)=B\left(t\right)e^{i\phi \left(t\right)} \end{array}$$

(3) Then, we calculated the relative phase between the two dancers' rhythmic movements using the phase data of each dancer.

$$\begin{array}{l} \Delta \phi_{AB}\left(t\right)=tan^{-1}\frac{P_{H_{A}}\left(t\right)\cdot P_{B}\left(t\right)-P_{H_{B}}\left(t\right)\cdot P_{A}\left(t\right)}{P_{A}\left(t\right)\cdot P_{B}\left(t\right)+P_{H_{A}}\left(t\right)\cdot P_{H_{B}}\left(t\right)} \end{array}$$

This relative phase of the rhythmic movement indicates the relationships of the upward and downward neck movements between two dancers. The relative phase at 0 degrees shows that the dancers moved in the same z-axis direction (i.e., if one dancer moved upward, the other also moved upward). The relative phase at −180/180 degrees shows that they moved in opposite directions (i.e., if one dancer moved upward, the other moved downward). Furthermore, we can investigate the leader-follower relationships between two dancers' movements by checking this relative phase. If the relative phases' frequencies at slightly over 0 (like 0–20 and 20–40 degrees) are high, the dancer who performs first leads their movements. If those slightly below 0 (like −20–0 and −40-−20) are high, the dancer who performs second leads their movements. (For all battles, we aligned the data of the first and second performers).

This study also generated the Virtual pair condition and compared the results of coordination with those of the Real pair condition (e.g., Bernieri et al., 1988; Dale et al., 2011). We calculated the relative phase in the Virtual pair by replacing the movement data of one dancer with that of the same dancer in other battles. We speculated that the battle scene rules and common inputs from the music beats could facilitate the coordination of the dancers' rhythmic movements. To calculate the baseline of the coordination generated by this kind of factor in the battle scenes, we generated the Virtual pairs and compared these results with those of the Real pairs.

## Results

### Relative Phase in the Whole Battle

Figure 2 shows representative examples of the dancer's individual movement data and the preprocessing practice. This figure shows that the dancers seemed to conduct vertical rhythmic movements throughout the battle scene, including before, during, and after the performance. The frequency spectrum showed a peak frequency at around 0.5, suggesting that the dancers participated in the battle by continuously conducting the rhythmic movements at about 2 Hz (Turn 1 in battles 1, 2). These results suggest that it is appropriate to use a relative phase analysis to investigate the dancers' interactions of a specific rhythmic movement in the battle.

Figure 3A shows the time series of the relative phase in the Real pair condition. This figure shows that the relative phase frequently indicated −180–−160 or 160–180 degrees, which suggests that the two dancers tended to adopt a rhythm in an anti-phase synchronization. They adopted a rhythm in the opposite pattern in the battle scene, especially in turns where the dancers showed their performances (painted in red and green in Figure 3A). However, as indicated in Figure 3B, we could not observe the same pattern of synchronization in the Virtual pair condition. The dancers in the Virtual pair condition tended to adopt a rhythm at various degrees, including 0–20 (in-phase synchronization), −180–−160, 160–180 (anti-phase synchronization), and other degrees, which means that they did not coordinate their movements in a specific pattern.

Figure 3C shows the frequency of each relative phase. The solid line in this figure confirms that the frequency of the relative phase at −180–−160 and 160–180 degrees were high in the Real pair condition, which means that the dancers coordinated their rhythmic movements in an anti-phase synchronization. However, the dotted line in Figure 3C shows that a similar tendency was not observed in the Virtual pair condition. In this condition, the frequencies of the relative phases at any specific degrees were not high. Two-way ANOVA revealed a significant interaction between the condition and the relative phase [*F*(17, 323) = 2.36, *p* < 0.01, η^2^ = 0.07]. *Post-hoc* analysis (simple main effect and multiple comparisons with Holm's correction) showed that in the Real pair condition, the frequencies of the relative phases at −180–−160 and 160–180 degrees were higher than those at other degrees [*F*(17, 136) = 23.42, *p* < 0.001, η^2^ = 0.74]. Significant differences were observed between the relative phase at−180-−160 degrees and each of −60–−40 (*p* < 0.05, *d* = 3.68), −40–−20 (*p* < 0.05, *d* = 4.24), −20–0 (*p* < 0.05, *d* = 4.63), and 0–20 degrees (*p* < 0.05, *d* = 3.78). They were also seen between 160–180 degrees and each of −60–−40 (*p* < 0.05, *d* = 3.77), −40–−20 (*p* < 0.05, *d* = 4.24), −20–0 (*p* < 0.05, *d* = 4.53), and 0–20 degrees (*p* < 0.05, *d* = 3.86). However, in the Virtual pair condition, the frequency of the relative phase at −180–−160 and 160–180 degrees was not higher than that of the other phases [*F*(17, 187) = 3.00, *p* < 0.001, η^2^ = 0.21, but *p* > 0.05 for all pairs between −180–−160 and other degrees and between 160–180 and other degrees; range of *d* = 0.01–1.44]. Furthermore, we assessed the differences of frequency between the two conditions. The frequency of the relative phase at 160–180 degrees in the Real pair condition was higher than that in the Virtual pair condition [*F*(1, 19) = 4.70, *p* < 0.05, η^2^ = 0.20], although at −180–−160 degrees, there was no significant difference between the conditions [*F*(1, 19) = 0.01, *p* = 0.48, η^2^ = 0.03].

### Relative Phase in Each Turn

**Figure 4:**
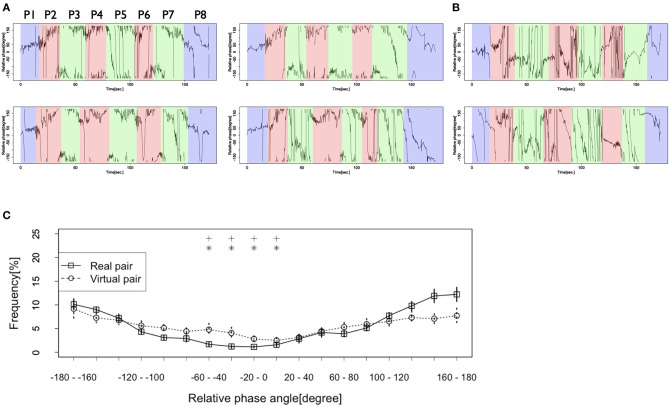
**(A)** Several examples of the relative phases in the Real pair condition. The spaces colored in blue show the time when both dancers did not show their performances (P1, P8), those colored by red show the performance time of the first dancer (P2, P4, P6), and those colored by green show the performance time of the second dancer (P3, P5, P7). **(B)** Several examples of the relative phases in the Virtual pair condition. **(C)** Frequencies of each relative phase of the dancers' rhythmic movements in the Real pair condition and the Virtual pair condition. Vertical lines at each plot indicate standard error. Asterisk and cross indicate the relative phases whose frequencies show significant differences with that at −180–−160 degrees and 160–180 degrees in the Real pair condition (*: *p* < 0.05 with −180–−160 degrees, +: *p* < 0.05 with 160–180 degrees).

**Figure 5:**
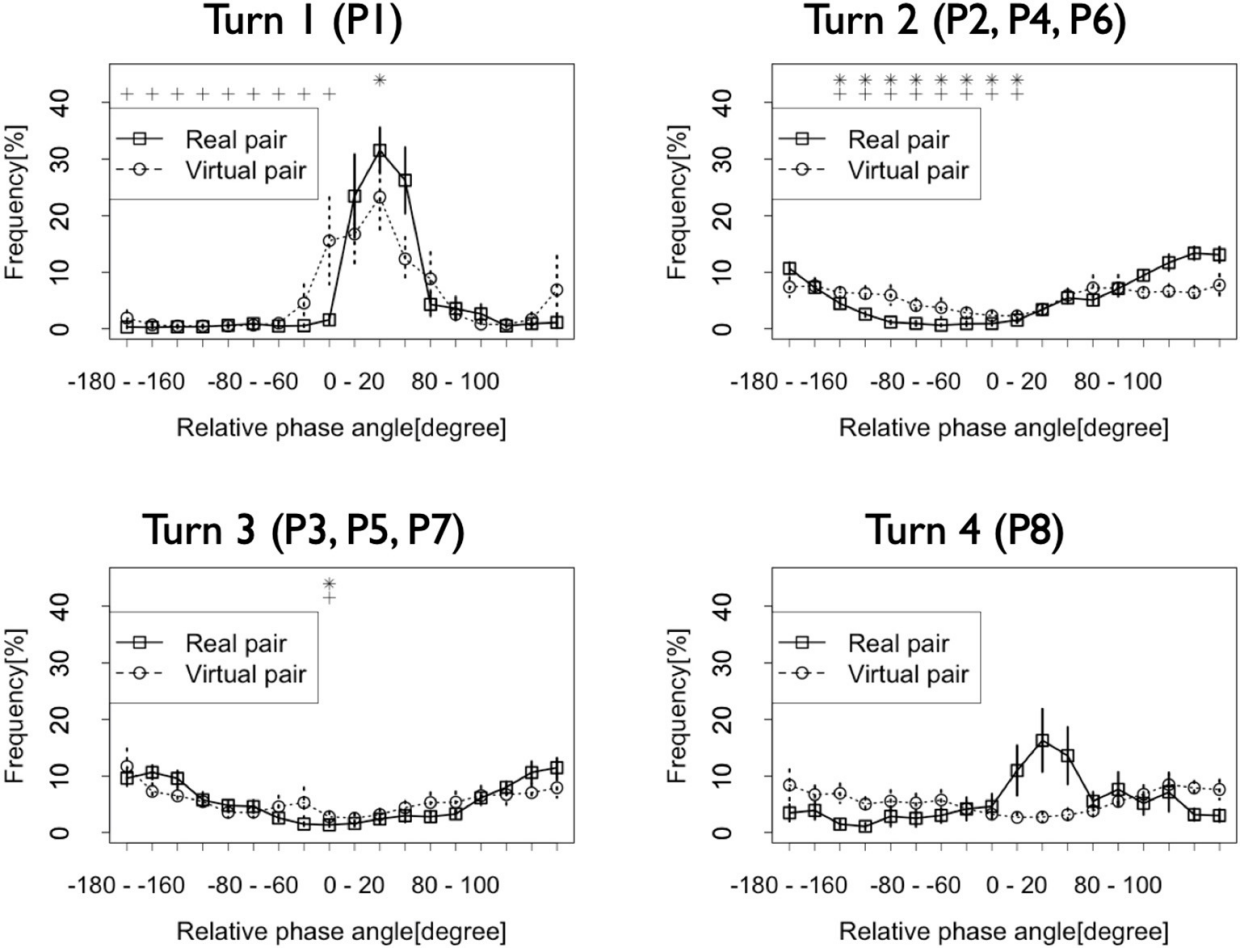
Frequencies of the relative phase of the dancers' rhythmic movements in each turn. Vertical lines at each plot indicate standard error. Asterisk and cross indicate the relative phases whose frequencies show significant differences with that at −20–0 degrees and 0–20 degrees in Turn 1 and Turn 4 (*: *p* < 0.05 with −20–0 degrees, +: *p* < 0.05 with 0–20 degrees) and −180–−160 degrees and 160–180 degrees in Turn 2 and Turn 3 (*: *p* < 0.05 with −180–−160 degrees, +: *p* < 0.05 with 160–180 degrees).

We also investigated the dynamic change in the patterns of this synchronization/coordination. Figure 4 shows the frequency of the relative phase in each turn. Turn 1 (P1 in Figure 5) was the time before both dancers started to show their performances. Turn 2 (P2, P4, P6) was the time when the first dancer showed his first, second, and third performances. Turn 3 (P3, P5, P7) was the time when the second dancer showed his first, second, and third performances. Turn 4 (P8) was the time after both dancers finished their performances. The solid lines in this figure indicate that in Turns 1 and 4, when neither dancer showed their performances, the relative phase frequencies at −180–−160 and 160–180 degrees were relatively low, and those at 0–20 degrees and 20–40 degrees were relatively high. However, in Turns 2 and 3, when the dancers showed their performances, the frequencies at −180–−160 and 160–180 degrees were high. On the other hand, those at −20–0, 0–20, and 20–40 degrees were relatively low. These results suggest that the dancers coordinated their rhythmic movements in an anti-phase synchronization only during the battle. Neither before the dancers started their performances nor after they finished their performances did they show the same synchronization pattern. In Turns 1 and 4, the frequencies at 0–20 and 20–40 degrees were relatively high. These results suggest that in these turns, they conducted their rhythmic movements in an in-phase synchronization. Additionally, the first dancer who started his performance just after Turn 1 tended to lead their rhythmic movements, and the second dancer followed him in these turns.

The outcomes of the statistical testing support these results. The results of the two-way ANOVA showed a significant interaction between the turn and the relative phase in the Real pair condition [*F*(51, 408) = 5.97, *p* < 0.001, η^2^ = 0.44]. *Post-hoc* analysis showed that in Turn 1, the frequency of the relative phase at 20–40 degrees was higher than that of the other phases [*F*(17, 136) = 18.46, *p* < 0.001, η^2^ = 0.68]. Significant differences were observed between the relative phase at 20–40 degrees and those at −180–0 and 100–180 degrees (all *p* < 0.05; range of *d* = 3.03–4.33). However, no significant differences were indicated between the phase at 0–20 degrees and other phases (all *p* > 0.05; range of *d* = 0.27–2.00). Additionally, in Turn 4, there were no significant differences among the phases [*F*(17, 136) = 2.35, *p* < 0.01, all pairs between 0–20 and other phases: *p* > 0.05, range of *d* = 0.10–1.45; all pairs between 20–40 and other phases: *p* > 0.05, range of *d* = 0.18–1.55]. In Turns 2 and 3, the frequencies at −180–−160 and 160–180 degrees were higher than those at other phases [Turn 2: *F*(17, 136) = 36.76, *p* < 0.001, η^2^ = 0.81; Turn 3: *F*(17, 136) = 12.08, *p* < 0.001, η^2^ = 0.59]. In Turn 2, significant differences were observed between the relative phase at −180–−160 degrees and −140–20 degrees (all *p* < 0.05; range of *d* = 2.44–5.53), and at 160–180 and −140–0 degrees (all *p* < 0.05; range of *d* = 2.88–5.67). In Turn 3, significant differences were observed between the relative phases at −180–−160 and −20–0 degrees (*p* < 0.05, *d* = 3.56), and at 160–180 and −20–0 degrees (*p* < 0.05, *d* = 3.51). These results suggest that in Turn 1, the first dancer frequently led their rhythmic movements, and in both Turns 2 and 3, the dancers took their rhythms in an anti-phase synchronization manner.

When we checked the differences among turns, we found that the frequencies of the relative phases at 0–20 and 20–40 degrees in Turn 1 were higher than those in other Turns [0–20: *F*(3, 24) = 9.27, *p* < 0.001, η^2^ = 0.40; 20–40: *F*(3, 24) = 15.90, *p* < 0.001, η^2^ = 0.57]. For 0–20 degrees, a significant difference was observed between Turns 1 and 2 (*p* < 0.05, *d* = 1.58). The difference between Turns 1 and 3 did not reach a significance, but it did have a large effect size (*p* = 0.05, *d* = 1.56). For 20–40 degrees, a significant difference was observed between Turn 1 and Turns 2 and 3 (Turn 1 and Turn 2: *p* < 0.001, *d* = 3.03; Turn 1 and Turn 3: *p* < 0.001, *d* = 3.26). Furthermore, the frequencies at −180–−160 and 160–180 degrees in Turns 2 and 3 were higher than those in other turns [−180–−160: *F*(3, 24) = 30.68, *p* < 0.001, η^2^ = 0.72; 160–180: *F*(3, 24) = 27.06, *p* < 0.001, η^2^ = 0.71]. For −180–−160 degrees, a significant difference was observed between Turn 1 and Turns 2, 3, and 4 (Turn 1 and Turn 2: *p* < 0.001, *d* = 5.75; Turn 1 and Turn 3: *p* < 0.001, *d* = 4.58; Turn 1 and Turn 4: *p* < 0.05, *d* = 1.23) and between Turns 2 and 3 and Turn 4 (Turn 2 and Turn 4: *p* < 0.05, *d* = 1.89; Turn 3 and Turn 4: *p* < 0.05, *d* = 1.59). For 160–180 degrees, a significant difference was observed between Turn 1 and Turns 2 and 3 (Turn 1 and Turn 2: *p* < 0.001, *d* = 3.77; Turn 1 and Turn 3: *p* < 0.01, *d* = 3.11) and between Turns 2 and 3 and Turn 4 (Turn 2 and Turn 4: *p* < 0.01, *d* = 2.59; Turn 3 and Turn 4: *p* < 0.05, *d* = 2.04).

These results support the observations made in Figure 4. Before their performances, the dancer who performed first frequently led their rhythmic movements. Additionally, they tended to coordinate their rhythms in an in-phase synchronization (though this is suggested only by the effect sizes). On the other hand, during their performances, they tended to conduct movements in an anti-phase synchronization. After their performances, the first dancer seemed to lead their rhythmic movements, though this is suggested only by the observation of the figure. It is not indicated by the results of the statistical tests.

## Discussion

This study showed that in the breakdance battle scene where two dancers competed against each other, they coordinated their rhythmic movements in an anti-phase synchronization. This result suggests the importance of focusing on the collaborative/competitive contexts when we investigate people's coordination in the performing arts. Studies investigating the performers' coordination are limited. Most of them have focused on the coordination in the collaborative context (e.g., Washburn et al., 2014; Weinstein et al., 2016; Walton et al., 2018). Performers' coordination in the competitive context has been frequently overlooked. However, in some genres of the performing arts, such as jazz sessions and breakdance battle scenes, performers frequently compete against each other and try to exhibit the superiority of their performances (Bailey, 1980; OHJI, 2001; Watkins, 2005). The performers' interaction in this context where they try to emphasize the individuality of their performances is an interesting phenomenon that requires additional scientific study. In addition, in a real performance scene, performers might interact with each other in a more complicated and mixed context of collaboration and competition, as a previous study suggested (Keller et al., 2017). We suppose that the Bass singers of the chorus in the above study experienced this mixed context. Further research is required that focuses on these context differences and that compares the differences in the performers' coordination brought on by these contexts.

We also need some discussions of the anti-phase synchronization of the performers' rhythmic movements observed in this study. This result is consistent with those of the previous studies investigating the coordination of people's behaviors in competitive conversations, such as debates (e.g., Paxton and Dale, 2013, 2017; Abney et al., 2014). These studies of competitive conversations showed the following results: (1) In a competitive context, the in-phase synchronization of people's behaviors, such as head movements, decreased. (2) The time-lagged coordination of people's behaviors was frequently observed. (3) A change in the time-lagged coordination structure like a change to the length of the time lag was observed. Moreover, the studies examining the behaviors of several insects in the competitive context have shown that they tend to chirp alternately, which has a similar structure to a time-lagged coordination (Jones, 1964; Walker, 1969). These results suggest that in the competitive context, people and also insects emphasize the features of their individual behaviors by generating some similarities (the frequency of movements) with, and some differences (the timing of movements) from, other individuals' behaviors simultaneously. Furthermore, in the breakdance battle scene, as the performers listened to the same music (OHJI, 2001; Watkins, 2005), we suppose that this environment facilitated the in-phase synchronization of people's behaviors as suggested by previous studies. We frequently observed this in-phase synchronization of the performers' rhythmic movements before they showed their performances (we had already started the music before the performances started). This result-the dancers showed the anti-phase synchronization in the in-phase facilitation environment-showed the essential aspects of people's behaviors in a competitive context. As discussed later, we consider that this coordination that generates similarities and differences simultaneously has the function of emphasizing the features of individual performances to the audience. However, to verify this function, we need to further investigate the audience's responses.

Furthermore, future research needs to focus on more complicated interactions that extend beyond simple coordination, such as in-phase synchronization and anti-phase synchronization. We confirmed the presence of a leader-follower coordination before the dancers' performances, with the dancer who performed first tending to lead their rhythmic movements. In this experiment, we did not decide the performance order in advance, and instructed the dancers to decide it nonverbally during this period. This setting is typical and well-known in the breakdance battle scene. We consider that this setting facilitated the aforementioned leader-follower coordination. The first dancer sent some signals to the second dancer to start the performance by emphasizing his rhythmic movement, and the second dancer reacted to it. Figure 3A also shows that just before the turn changes, the dancer who performed next tended to lead their rhythmic movements. Furthermore, we observed the leader-follower coordination after the battle was completed. We can explain this by the fact that the battles have ended at the first dancer's preparation timing, although they both knew the number of turns. The above leader-follower coordination suggests that a more complex phenomena beyond the simple coordination can occur in battle scenes. (We can list a polyrhythm as an example that we could not capture in this study). Furthermore, based on the background of the dance movements and the results of the individual movements, dancers would actively interact with each other by using several expression mediums, such as rhythmic movements, dance steps, gestures, and facial expressions (Shimizu and Okada, 2013). To comprehensively investigate the complexity of the performers' interactions in the battle scene, we would need to capture the coordination from the viewpoints of multiple mediums (Louwerse et al., 2012) and multiple scales (Zapata-Fonseca et al., 2016). Several studies propose a framework called *beyond synchrony* that tries to develop a synchronization theory so as to capture people's complicated coordination in everyday conversations and joint actions (e.g., Dale et al., 2013; Wallot et al., 2016). Based on this framework and careful observation of the domains, we can develop a framework by which to capture the complicated coordination among people in the performing arts. Complexity matching and the Allan Factor suggested by Zapata-Fonseca et al. (2016) are representative examples of this framework development and analysis.

Finally, we need further studies to investigate the pro-social effect of these patterns of coordination in the competitive context. Past studies in social psychology have demonstrated that in-phase synchronization facilitates pro-social behaviors among people and strengthens their relationships (Hove and Risen, 2009; Wiltermuth and Heath, 2009). Several studies have confirmed that the same pro-social effect of in-phase synchronization occurs in music and dance performances (Kirschner and Tomasello, 2010; Weinstein et al., 2016). Moreover, some studies have suggested that because of this function, music and dance performances have spread across almost all cultures and societies (Fitch, 2006; Merker et al., 2009, 2015). However, regarding the pro-social effect of the anti-phase synchronization, past studies have not been able to show consistent results. Some studies have confirmed the effect (Cirelli et al., 2014; Sullivan et al., 2014), but other studies have not (Cross et al., 2016). Additionally, we need further investigations of the pro-social effect of the dynamic changes in coordination that our study suggested. Notably, in the history of breakdance, gangs have used breakdance as an alternative to fighting (OHJI, 2001; Watkins, 2005). It has been suggested anecdotally that the battle performance has a function of improving relationships among opposing gangs. The above discussion of the pro-social effect of coordination in the competitive context might be connected with this history of breakdance.

Several caveats must be addressed. First, the number of dancers in this study was limited. It was very difficult to conduct this group experiment, which featured busy expert dancers. Given these circumstances, we discussed the results of the experiment while focusing on both effect sizes and tests for significance. However, to widely generalize this study's results, it is essential to undertake further investigation with a larger number of expert dancers. Second, it will also be essential to set conditions that strongly reflect the contexts of coordination and competition. The present study investigated the context's influence by comparing the battle phases, before, after, and during the performance. However, to detect any differences more clearly in the effects of context, we need to compare conditions that directly reflect competitive and cooperative contexts. To compare the dancers' interaction between the battle scene and the cipher scene that is a more cooperative famous scene in the domain is a good example. Finally, it will be essential to undertake further studies that investigate the relationship between the music and dancers' movements. The current study detected the dancers' interactions specifically by generating the Virtual pairs. However, we could not investigate the music's function and role, which exist as common battle scene inputs. It will be necessary to develop and apply methods by which to analyze the interaction between different mediums like music and movement. By undertaking such efforts, it will be possible to comprehensively capture the interactions within a battle scene involving multiple participants who have different communication mediums. (The DJ selects and edits the music in the battle scene.) Performer coordination in the competitive context is an exciting subject that requires much more scientific investigation.

## Data Availability Statement

The raw data supporting the conclusions of this article will be made available by the corresponding author.

## Ethics Statement

The studies involving human participants were reviewed and approved by The University of Tokyo. The patients/participants provided their written informed consent to participate in this study.

## Author Contributions

DS performed the experiment, analysis, and interpretation. DS drafted the manuscript and TO provided critical revisions. All authors contributed to the study design and approved the final version of the manuscript for submission.

## Conflict of Interest

The authors declare that the research was conducted in the absence of any commercial or financial relationships that could be construed as a potential conflict of interest.
